# Establishment of a pseudovirus neutralization assay for TGEV

**DOI:** 10.3389/fimmu.2025.1558604

**Published:** 2025-04-10

**Authors:** Haojie Wang, Jianxing Chen, Lihong Xue, Yue Sun, Tongqing An, Yue Wang, Hongyan Chen, Changqing Yu, Changyou Xia, He Zhang

**Affiliations:** ^1^ State Key Laboratory for Animal Disease Control and Prevention, Harbin Veterinary Research Institute, Chinese Academy of Agricultural Sciences, Harbin, China; ^2^ School of Advanced Agricultural Sciences, Yibin Vocational and Technical College, Yibin, China

**Keywords:** TGEV, pseudovirus, neutralizing antibody, pseudovirus neutralization test, ST cells

## Abstract

**Introduction:**

Transmissible Gastroenteritis Virus (TGEV) is a major pathogen causing swine enteric diseases, necessitating effective control strategies. Vaccination plays a key role, but assessing vaccine efficacy remains challenging due to variations in immune response and existing detection limitations. Current antibody detection methods, such as neutralization assays and ELISA, are often subjective, labor-intensive, and time-consuming, highlighting the need for a more efficient evaluation approach.

**Methods and results:**

The TGEV S gene was amplified and inserted into the eukaryotic vector PM2.G-ΔG-HA to construct the recombinant plasmid PM2.G-ΔG-TGEV-S-HA. Transfecting ST cells with this plasmid, followed by infection with G*VSV-GFP/LUC, successfully produced TGEV P0 pseudoviruses. Western blot and electron microscopy confirmed the presence of TGEV S and VSV N proteins and the distinct pseudovirus morphology. Optimization determined that 0.5 μg/well of plasmid, 24 h transfection, and 24 h post-infection harvest yielded a viral titer of 10^6^-10^7^ TCID_50_/mL. The pseudoviruses exhibited strong ST cell tropism and were effectively neutralized by TGEV-positive sera. A pseudovirus-based neutralization test (pNT) was established, showing 100% sensitivity, 96.6% specificity, no cross-reactivity with PEDV, PPV, PDCoV, or PRoV, and a 94% concordance with the live virus neutralization test. The method effectively tracked antibody level changes post-TGEV vaccination.

**Discussion:**

This study successfully developed a novel pseudovirus-based detection method, overcoming traditional assay limitations. The pNT method provides a scalable, efficient, and reliable tool for TGEV antibody evaluation, with broad potential applications in pathogen detection and vaccine assessment.

## 1 Introduction

In recent years, coronavirus (CoV)-induced infectious diseases have caused significant economic losses to global livestock industries and posed critical public health challenges (1, 2). Among these, transmissible gastroenteritis (TGE), caused by the transmissible gastroenteritis virus (TGEV) in the family *Coronaviridae*, is an acute, highly contagious enteric disease primarily affecting pigs, especially suckling piglets (3, 4). The clinical manifestations of TGE include vomiting, severe diarrhea, and dehydration, with rapid disease progression and mortality rates exceeding 90% in piglets, resulting in substantial economic losses annually (5). Recognized as a major animal disease of global concern, TGE has been classified as a List B disease by the World Organisation for Animal Health (WOAH) (6). Since its first identification in the 1930s, TGEV has been widely reported in intensive pig farming regions across the Americas, Europe, and Asia (7). The virus was initially detected in Taiwan Province of China in 1958 and continues to sporadically emerge in various regions of China, posing a significant threat to the global swine industry (8). TGEV is an enveloped, single-stranded positive-sense RNA virus (9). The viral particles exhibit pleomorphism under electron microscopy, with most appearing spherical or elliptical in shape, and have a diameter ranging from approximately 90 to 200 nm. The surface of the envelope is adorned with petal-like spikes (10). The TGEV genome is 28.6 kb in length and includes a 5′ leader sequence, a 200-bp untranslated region (UTR), a 3′ UTR, and nine open reading frames (ORFs) (11). ORF1a and ORF1b occupy two-thirds of the genome and are responsible for genome replication, while ORFs 2, 4, 5, and 6 encode the spike (S), nucleocapsid (N), membrane (M), and envelope (E) proteins, respectively, which collectively form the structural framework of the virus (9). Among these, the S protein plays a pivotal role in mediating viral entry by binding to host cell receptors and inducing neutralizing antibody production (12, 13). The S protein is thus a prime target for the development of TGEV vaccines and molecular diagnostic assays.

Accurate diagnostic tools are essential for the detection and control of TGE (14, 15). Immunofluorescence assays offer high sensitivity but require sophisticated equipment and expertise, restricting their use to research laboratories (16, 17). Serological methods like enzyme-linked immunosorbent assays (ELISA) are simple and reliable, widely applied in disease surveillance and product development, although they depend on high-quality samples and optimal conditions (18). Rapid detection techniques such as colloidal gold tests are field-friendly but constrained by sample concentration requirements (19). Neutralization assays uniquely combine precision in diagnosing viral immunity with vaccine efficacy evaluation. These assays, regarded as the gold standard, include plaque reduction neutralization tests and microneutralization tests (20–23). However, due to TGEV’s high transmissibility and environmental stability especially in low-temperature and high-humidity conditions the use of live virus poses a significant biohazard (7, 24).

Pseudoviruses (PsVs) provide a safe and efficient alternative for serological neutralization tests (25). As chimeric viral particles constructed through the integration of envelope or capsid proteins with nucleic acids, pseudoviruses lack replication capability, ensuring high biosafety and allowing their use in low-containment laboratories (26). This unique property makes them invaluable for studying viral infection mechanisms, host interactions, cell tropism, and entry pathways, as well as for applications in neutralization assays, vaccine evaluations, and antiviral drug screening (27–29). Similar to this, Lambda phage has been utilized in research areas such as gene cloning and expression, genomic studies, gene transfer, and vaccine delivery. However, there have been no reports on its use for the establishment of neutralizing antibody detection methods (30, 31).

To mitigate the risks associated with handling live TGEV, we utilized the vesicular stomatitis virus (VSV) pseudovirus system to construct a TGEV pseudovirus. The TGEV S gene was recombined into the VSV backbone plasmid and transfected into target cells. Using the G*VSV-ΔG-GFP/LUC system, we successfully packaged pseudovirus particles displaying TGEV-specific surface antigens. This pseudovirus was employed to develop a pseudovirus-based neutralization assay (PsV-NA), enabling the evaluation of serum neutralization capacity against TGEV in a simplified and biosafe manner. This assay provides a reliable alternative for determining neutralizing antibody titers without the risks associated with live-virus manipulation. Furthermore, it offers a robust platform for TGEV vaccine evaluation and clinical diagnostics, with significant implications for disease control and prevention.

## 2 Materials and methods

### 2.1 Plasmids, genomes, and cells

The G*VSV-ΔG-GFP and G*VSV-ΔG-LUC pseudovirus packaging system was generously provided by the National Institutes for Food and Drug Control (Beijing, China); the TGEV genome, TGEV-negative serum, and TGEV-positive serum were kindly provided by the Innovative Research Team for Swine Digestive Tract Infectious Diseases at Harbin Veterinary Research Institute. The HA antibody was purchased from Abmart Biopharma (Shanghai, China), and the VSV N protein antibody was obtained from Beijing Sino Biological Technology Co., Ltd. The ST cells (porcine testicular cells), PK-15 cells (porcine kidney cells), BHK21 cells (hamster kidney cells), HEK 293T cells (human embryonic kidney cells), positive serum of PEDV, PPV, PDCoV, and PRoV (All were validated using classical methods.) were preserved in our laboratory.

### 2.2 Construction and validation of the recombinant TGEV S gene plasmid

Based on the TGEV S gene sequence (GenBank: DQ811788.1), primers were designed using Primer Premier 5 software and synthesized by Sangon Biotech (Shanghai) Co., Ltd. (**Table 1**). The S gene fragments were amplified by RT-PCR and fused into the full-length S gene. The fusion PCR product was purified using a FastPure Gel DNA Extraction Mini Kit (Vazyme, China) and then ligated into PM2.G-ΔG-HA (Shanghai Lupubio Co., Ltd, China) according to the recommended reaction system and conditions in the ClonExpress Ultra One Step Cloning Kit (Vazyme, China) manual, resulting in the recombinant plasmid PM2.G-ΔG-TGEV-S-HA. The plasmid was then transformed into *DH5α* competent cells, followed by shaking at 37°C for 1 hour for recovery. The cells were plated on LB agar medium and incubated upside down at 37°C for 12-16 h. Colonies were screened, and plasmids were extracted and verified by PCR, double digestion with restriction enzymes *EcoR*I and *Xho*I, and sequencing. Verified colonies were inoculated in bulk culture at a ratio of 1:1000. The recombinant plasmid was extracted using a plasmid extraction kit. Seeded 293T cells in a 12-well plate 24 hours in advance. When the cell density reached 80-90%, 1 μg of the recombinant plasmid PM2.G-ΔG-TGEV-S-HA was transfected into Human Embryonic Kidney 293T cells using X-tremeGENE™ HP DNA Transfection Reagent (Merck, Germany), following the manufacturer’s instructions. After 24 hours, collected the cell samples, centrifuged at 12,000 rpm for 2 minutes, discarded the supernatant, and retained the pellet. The supernatant was discarded, and the pellet was retained. The harvested sample was resuspended in 60**-**100 μL of cell lysis buffer, mixed using a vortex mixer or pipette, and lysed on ice for 30 minutes. After lysis, the cell lysate was centrifuged, and the supernatant was transferred to a new 1.5 mL microcentrifuge tube. Next, 15**-**20 μL of 6× Protein Loading Dye was added to the supernatant, mixed thoroughly, centrifuged briefly, and heated in a 100°C heat block for 10 minutes. The expression of the TGEV S protein was then validated by Western blot analysis.

**Table 1:** Primers for amplifying the TGEV S gene sequence.

Primer	Sequence (5′to 3′)	Size (bp)
TGEV-S1-F	ATGAAAAAATTATTTGTGGTTTTGGT	883
TGEV-S1-R	cggctgtttggtaaCTAATTTACCACTAACCAACGTGGA	
TGEV-S2-F	aattagTTACCAAACAGCCGTTATTAGTTAATT	1235
TGEV-S2-R	gcgtcctgttagtttgtctaATAATACCAACACCAGTTCTACCATATATATT	
TGEV-S3-F	TAGACAAACTAACAGGACGCTACTTAGTG	974
TGEV-S3-R	gggatgctgtgtaCATAGTCATTTTGTCAGCATTAGCC	
TGEV-S4-F	gactatgTACACAGCATCCCTCGCAGG	1111
TGEV-S4-R	tgagactgagaCCTAACGCATTCATTAACCTTGTC	
TGEV-S5-F	tgcgttaggTCTCAGTCTCAGAGATTCGGATTCT	1253
TGEV-S5-R	TTAATGGACGTGCACTTTTTCAA	
TGEV-S-F	ATCATTTTGGCAAAGAATTCGCCACCATGAAAAAGCTGTTCGTGG	4344
TGEV-S-R	ACATCGTATGGGTACTCGAGGTGCACATGCACCTTTTCAATAGGC	

### 2.3 Construction and validation of TGEV S pseudovirus

Pre-seeded 293T cells in a T75 cell culture flask for proliferation. When the cell density reached 80-90%, 12 μg of the recombinant plasmid PM2.G-ΔG-TGEV-S-HA was transfected into the cells using X-tremeGENE™ HP DNA Transfection Reagent (Merck, Germany), following the manufacturer’s instructions. After 24 h, inoculated the cells with G*VSV-ΔG-GFP pseudovirus (32). Following a 24-hour infection period, the viral supernatant was harvested and filtered through a 0.45 μm filter to obtain the P0 generation of TGEV pseudovirus. The P0 TGEV pseudovirus was incubated with VSV G protein antibodies and then inoculated into ST and PK-15 cells for fluorescence observation to confirm whether the TGEV S pseudovirus could infect the target cells. Subsequently, the P1 generation of TGEV S pseudovirus (P1 PsV) was amplified. Cell precipitates and supernatants were separated, with the supernatants filtered through a 0.45 μm filter and concentrated by ultracentrifugation at 72,000 g for 3 h. Both the concentrated virus and the cell precipitates were validated using Western blot analysis. Finally, the morphology of the P1 TGEV PsV was observed using transmission electron microscopy to confirm the successful packaging of the TGEV pseudovirus.

### 2.4 Optimization of TGEV S pseudovirus packaging conditions and titer determination

To produce high-titer TGEV S pseudovirus, several parameters were screened and optimized: the transfection dose of recombinant plasmid PM2.G-ΔG-TGEV-S-HA (0.1 μg/well, 0.5 μg/well, 1 μg/well, 1.5 μg/well, and 2 μg/well), transfection time (12 h, 24 h, 36 h, and 48 h), target cells (ST cells, PK-15 cells, BHK21 cells, and 293T cells), and virus harvest time (12 h, 24 h, 36 h, and 48 h). The relative luminescence unit (RLU) values were measured using a multifunctional microplate reader to compare viral titers under different conditions. The optimized TGEV P0 generation pseudovirus (P0 PsV), treated with VSV G protein, was subjected to 10-fold serial dilutions and inoculated into ST cells in a 96-well plate. After 24 h of incubation, the RLU values were measured (Determine the RLU value according to the instructions provided in the kit’s manual). Wells with luminescence values three times higher than the negative control were considered positive. The 50% tissue culture infectious dose (TCID_50_) of the pseudovirus was calculated using the classical Reed-Muench method (33).

### 2.5 Establishment of TGEV S pseudovirus neutralization test

To further validate the neutralization activity of the TGEV S pseudovirus, TGEV-positive and negative sera were subjected to 2-fold serial dilutions and mixed with the TGEV S pseudovirus for neutralization testing. Specifically, the TGEV-positive and negative sera were serially diluted (1:2, 1:4, 1:8, 1:16, 1:32, 1:64, 1:128, 1:256, 1:512, 1:1024, and 1:2048), and equal volumes of TGEV S pseudovirus were added at a 1:1 ratio. The serum-pseudovirus mixtures were incubated at 37°C for 1 hour to allow antibodies in the sera to bind to the pseudovirus. The incubated serum-pseudovirus mixtures were then inoculated onto pre-seeded ST cells. Each dilution gradient was tested in triplicate to ensure the reliability of the results. The cells were incubated at 37°C with 5% CO_2_ for 24 to 48 h, during which the cells absorbed and were infected by the pseudovirus. The relative luminescence unit (RLU) values of each group were measured using a chemiluminescence or fluorescence detector to assess the neutralization effects. Lower RLU values indicated stronger neutralization activity of the antibodies in the sera, while higher RLU values suggested insufficient antibodies or ineffective neutralization. By comparing the changes in RLU values across the dilution gradients, the neutralization efficiency of TGEV-positive sera was systematically evaluated. The neutralization characteristics of the pseudovirus and the reliability of the experiment were also confirmed through these comparisons.

### 2.6 Optimization of TGEV S pseudovirus neutralization test conditions

To achieve the optimal neutralization test conditions, factors influencing the test were optimized, including detection time (12 h, 24 h, 36 h, 48 h, 60 h, and 72 h), cell seeding density (1×10^4^, 2×10^4^, 3×10^4^, 4×10^4^, 5×10^4^, 6×10^4^, 7×10^4^, 8×10^4^, and 9×10^4^ cells/well), and pseudovirus concentration (50 TCID_50_/50 μL, 100 TCID_50_/50 μL, 200 TCID_50_/50 μL, 400 TCID_50_/50 μL, 800 TCID_50_/50 μL, and 1600 TCID_50_/50 μL). The RLU values were measured and compared to determine the optimal conditions for the TGEV S pseudovirus neutralization test.

### 2.7 Sensitivity, specificity, and stability validation

To assess the sensitivity, specificity, and stability of the TGEV S pseudovirus neutralization test, we selected 60 pig serum samples with well-defined backgrounds (30 negative and 30 positive sera) and tested them using the pseudovirus-based neutralizing antibody detection method established in this study. Sensitivity and specificity were analyzed using SPSS software and GraphPad Prism 8. Subsequently, serum samples from pigs infected with TGEV (titer 1:512), PEDV (titer 1:256), PPV (titer 1:1024), PDCoV (titer 1:512), and PRoV (titer 1:512) were tested to verify whether cross-reactivity occurred with positive sera from these viruses. Lastly, three TGEV-positive serum samples were tested in parallel by three different operators. Each operator conducted three replicate experiments using the same serum sample to ensure consistency and reliability. Repeat tests were performed once a week for three weeks, resulting in a total of nine tests for each serum sample, covering intra-batch repeatability and inter-operator comparability. The RLU values of the same serum sample were compared across different time points and operators to assess the reproducibility of the method under varying conditions.

### 2.8 Comparison of TGEV S pseudovirus neutralization assay with existing methods

To validate the accuracy of the TGEV S pseudovirus neutralization assay established in this study, we compared it with a commercial TGEV blocking ELISA antibody detection kit and the TGEV live virus neutralization assay. The TGEV S pseudovirus neutralization test and the commercial TGEV blocking ELISA kit were used to test 19 serum samples, and statistical methods were employed to assess the consistency of their results. Additionally, the newly established detection method was used in parallel with the TGEV live virus neutralization assay to test 50 pig serum samples, comparing the concordance of positive/negative results and serum titers.

### 2.9 Vaccine efficacy monitoring

To evaluate whether the TGEV S pseudovirus neutralization assay can be used for vaccine efficacy assessment, we collected 5 serum samples at different time points (0, 14, 28, 42, 56, 70, and 84 days) from pigs immunized with the TGEV vaccine, as well as 5 serum samples from pigs that were not vaccinated. Both the TGEV S pseudovirus neutralization test and the TGEV live virus neutralization assay were used to analyze the samples. This comparison aimed to determine whether the TGEV S pseudovirus neutralization assay can replace the live virus neutralization assay and effectively reflect the changes in antibody levels following vaccination.

### 2.10 Western blot analysis

Prepared protein samples were loaded into the wells of an SDS-PAGE gel. Electrophoresis was performed at 90 V for 30 minutes, then adjusted to 130 V until the samples reached the bottom of the gel. The PVDF membrane was activated and soaked in transfer buffer along with filter paper. After ensuring no air bubbles, the gel and membrane were assembled for transfer. The transfer was carried out at 15 V for 50 minutes. The membrane was blocked for 1 hour and washed 3-4 times with PBST. Primary antibodies (e.g., anti-HA or actin antibodies) were incubated with the membrane for 2 h, followed by washing and incubation with HRP-conjugated secondary antibodies for 45 minutes to 1 hour. After washing, the membrane was scanned using a near-infrared fluorescence imaging system to save the images.

## 3 Results and analysis

### 3.1 Construction and verification of the recombinant plasmid expressing TGEV S protein

**Figure 1:**
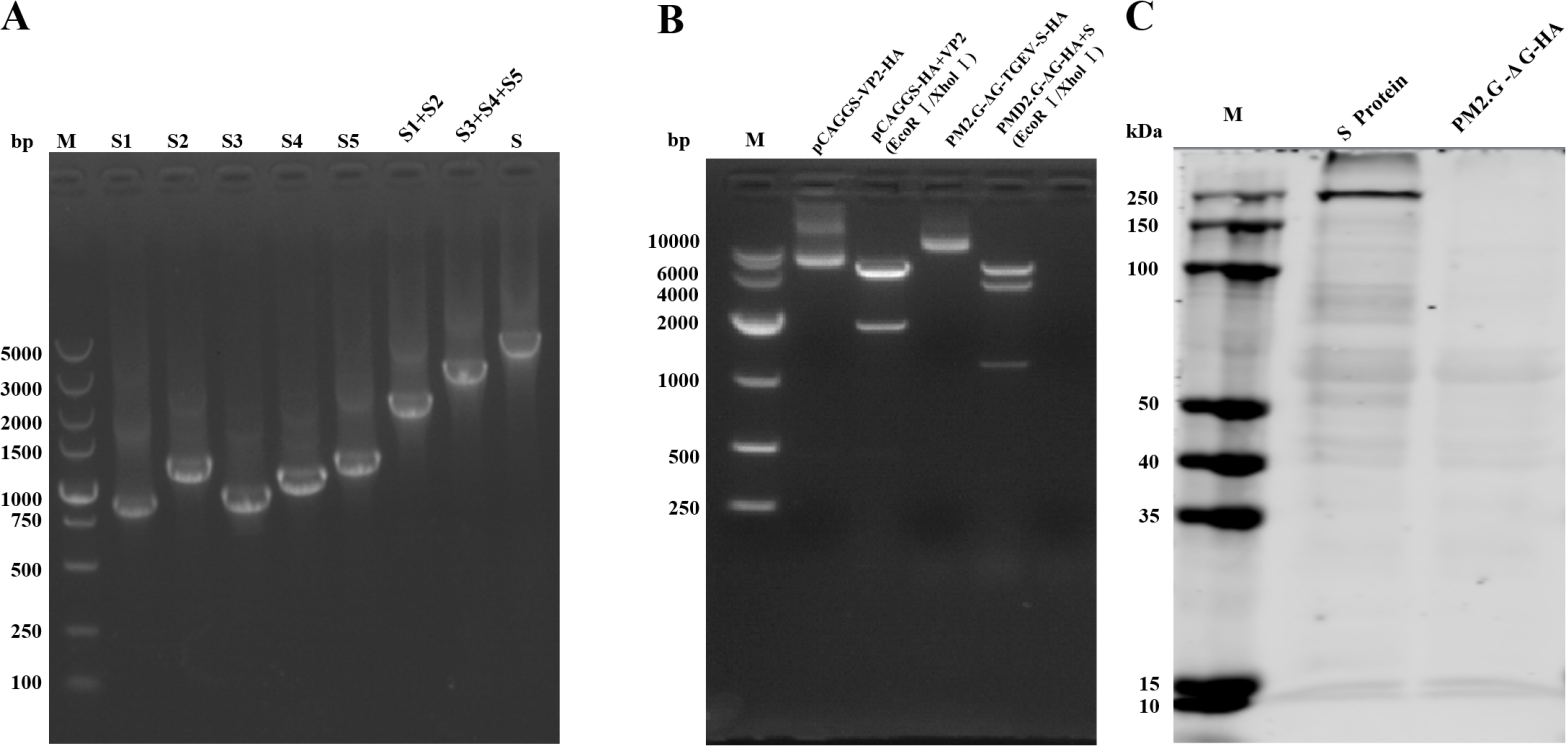
Construction of the recombinant plasmid containing the TGEV S gene and validation of S protein expression. **(A)** PCR amplification of the TGEV S gene. M: 5000bp DNA marker. S1-S5: Partial fragments of TGEV S gene (883, 1235, 974, 1111, 1253 bp). Lane 7-8: Partial fragments of TGEV S gene linked by fusion PCR. S: The full length of TGEV S gene (4344 bp). **(B)** Double-enzyme digestion (*EcoR*I and *Xho*I) of the recombinant plasmid PM2.G-ΔG-TGEV-S-HA. M: 10000bp DNA marker. Lane 3 is the full-length recombinant plasmid PM2. G-ΔG-TGEV-S-HA. Lane 4 shows the double enzyme digestion result of recombinant plasmid PM2. G-ΔG-TGEV-S-HA. **(C)** Validation of TGEV S protein expression. M: Prestained Protein Marker 10-250 kDa. Lane 3-4 shows the results of TGEV positive serum detection after transfecting plasmids PM2. G-ΔG-TGEV-S-HA and PM2. G-ΔG-HA into cells, respectively.

To construct the recombinant plasmid expressing TGEV S protein, we amplified the TGEV S gene fragment using fusion PCR. The obtained fragment was 4344 bp in length (**Figure 1A**). This fragment was then cloned into the plasmid PM2.G-ΔG-HA, resulting in the recombinant plasmid PM2.G-ΔG-TGEV-S-HA. Next, the recombinant plasmid was subjected to double digestion with *EcoR*I and *Xho*I restriction enzymes to verify the insertion of the TGEV S gene fragment. The digestion results, shown in **Figure 1B**, revealed two expected fragments: one corresponding to the vector (5975 bp) and the other to the S gene fragment (4344 bp), confirming the successful insertion of the TGEV S gene into the plasmid. Additionally, sequencing analysis was performed to further confirm the correctness of the recombinant plasmid. The sequencing results were consistent with the expected sequence, further validating the accuracy of the plasmid construction. To determine whether the S gene could be successfully expressed in the host cells, we transfected the recombinant plasmid PM2.G-ΔG-TGEV-S-HA into 293T cells. After 24 h of transfection, cells were collected and subjected to Western blotting using positive serum from TGEV-infected animals. In the Western blot experiment, a specific band around 220 kDa was detected in lane 1 (**Figure 1C**), which corresponds to the molecular weight of the TGEV S protein. This result confirms that the S gene was successfully expressed in 293T cells.

### 3.2 Packaging and verification of TGEV pseudovirus

**Figure 2:**
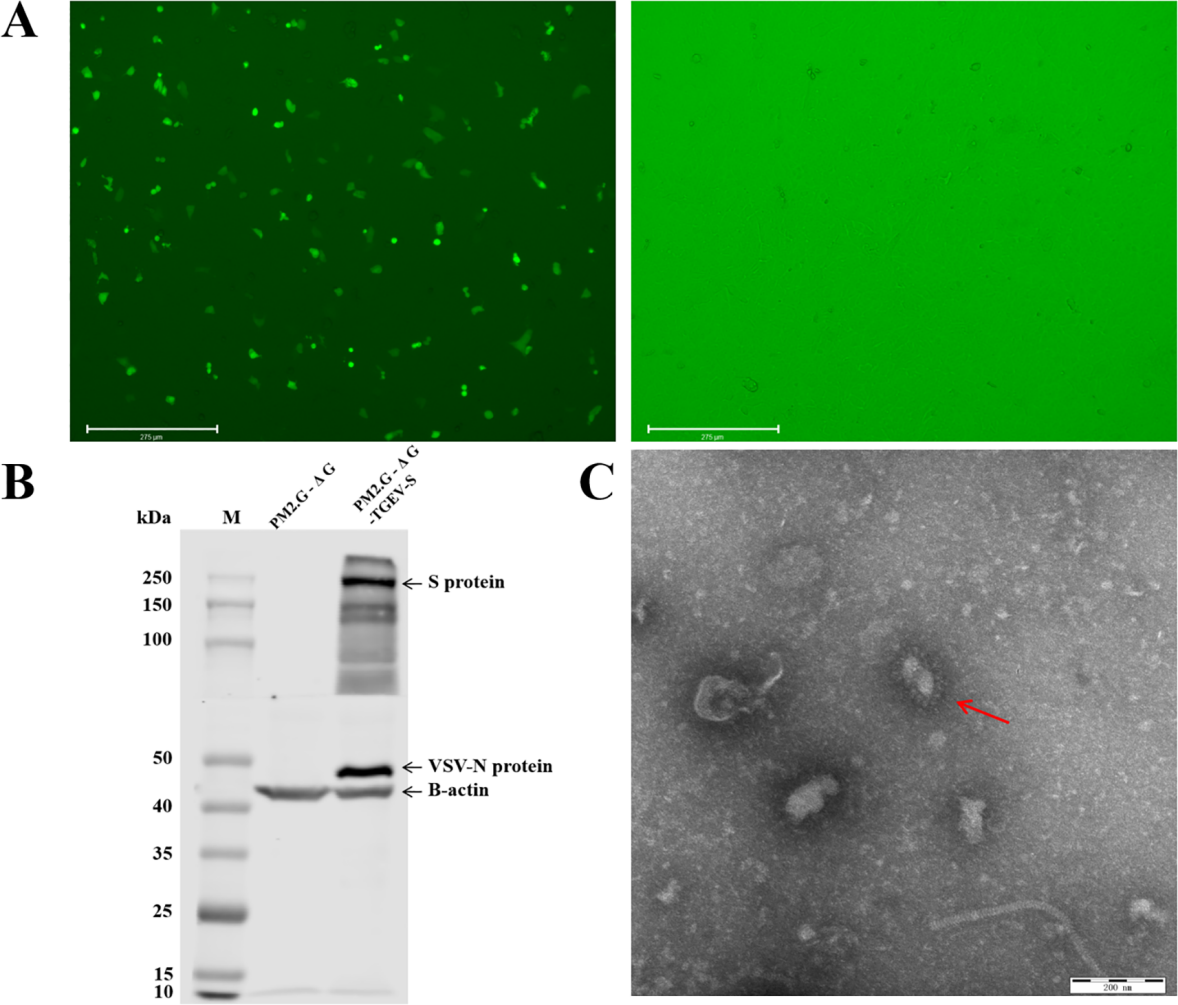
Packaging and validation of TGEV pseudovirus. **(A)** Package the pseudovirus using ST cells. Transfection of recombinant plasmids PM2. G-ΔG-TGEV-S-HA and PM2. G-ΔG-HA using fluorescence inverted microscopy, and infection with G*VSV-GFP pseudovirus. **(B)** Western blot validation of TGEV pseudovirus. M: Prestained Protein Marker 10-250 kDa. Lane 3-4 shows the results of TGEV positive serum and VSV N protein antibody detection after transfecting plasmids PM2. G-ΔG-HA and PM2. G-ΔG-TGEV-S-HA into cells and infecting them with G virus, respectively. **(C)** Transmission electron microscopy images of TGEV pseudovirus.

To prepare TGEV S pseudovirus, we first transfected the recombinant plasmid PM2.G-ΔG-TGEV-S-HA into 293T cells and utilized the G*VSV-ΔG-GFP pseudovirus system to generate P0 TGEV S pseudovirus. After 24 h of transfection, we incubated the P0 TGEV S pseudovirus with VSV G protein antibody to neutralize any residual G*VSV-ΔG-GFP pseudovirus. After neutralization, the P0 pseudovirus was inoculated into ST and PK-15 cells, and fluorescence microscopy was performed. The results showed strong green fluorescence in the ST cells, indicating that the TGEV S pseudovirus successfully entered the cells and replicated, generating P1 pseudovirus (**Figure 2A**). In contrast, the G*VSV-ΔG-GFP pseudovirus, which did not package TGEV S protein, was unable to replicate within the cells and showed no fluorescence.

To further confirm the correct insertion of the TGEV S protein, we performed Western blot analysis using TGEV-positive serum and a specific antibody against VSV N protein on P1 TGEV S pseudovirus. The Western blot results, shown in **Figure 2B**, revealed bands for both TGEV S protein and VSV N protein in the P1 TGEV S pseudovirus lane, while no such bands were detected in the G*VSV-ΔG-GFP pseudovirus lane. This indicates that the TGEV S protein was successfully inserted and correctly expressed in the pseudovirus.

To ensure the integrity and structure of the pseudovirus, we concentrated the P1 TGEV S pseudovirus and observed its particle morphology using transmission electron microscopy (TEM). The TEM images (**Figure 2C**) showed that the TGEV S pseudovirus particles had a diameter of approximately 200 nm and exhibited a typical bullet-shaped morphology with prominent spike structures on their surface, resembling the structural characteristics of the natural TGEV virus. These results demonstrate that the TGEV S pseudovirus not only effectively expresses TGEV S protein functionally but also exhibits a morphology and structure similar to that of the natural TGEV virus, confirming the successful construction of the pseudovirus system.

### 3.3 Optimization of packaging conditions for TGEV S pseudovirus

**Figure 3:**
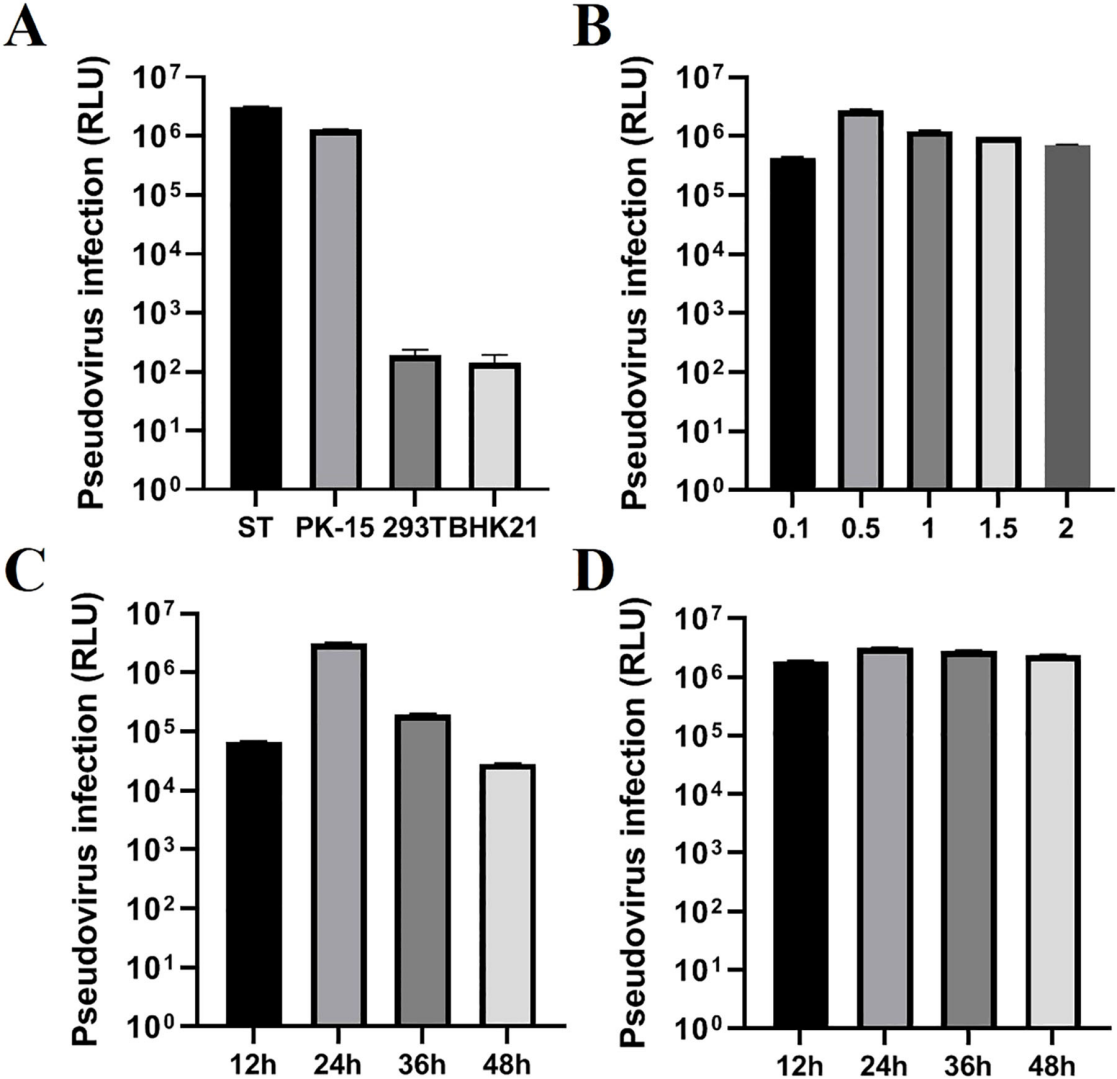
Optimization of TGEV pseudovirus packaging conditions. **(A)** Selection of the cell line (ST, PK-15, 293T, BHK21), testing four types of cells for TGEV pseudovirus. **(B)** Optimization of the transfection dose (0.1, 0.5, 1.0, 1.5, 2.0) of recombinant plasmid PM2.G-ΔG-TGEV-S-HA. **(C)** Optimization of the transfection time (12, 24, 36, 48h) for recombinant plasmid PM2.G-ΔG-TGEV-S-HA; **(D)** Optimization of the collection time (12, 24, 36, 48h) for TGEV P0 pseudovirus.

To obtain a high titer of TGEV S pseudovirus, we optimized the transfection conditions for the recombinant plasmid PM2.G-ΔG-TGEV-S-HA, including factors such as transfection dosage, transfection time, P0 TGEV S pseudovirus harvest time, and cell tropism. First, we screened for the most suitable target cell type. The results showed that ST cells exhibited a higher tropism for TGEV S pseudovirus packaging and replication (**Figure 3A**). After determining the optimal cell type, we continued to optimize the transfection dosage of the recombinant plasmid PM2.G-ΔG-TGEV-S-HA. Experimental screening indicated that a transfection dose of 0.5 μg/well produced the highest pseudovirus titer without significant cytotoxicity (**Figure 3B**). Next, we optimized the transfection time, and the results showed that TGEV S pseudovirus expression peaked 24 h post-transfection, with ideal transfection efficiency (**Figure 3C**).

Additionally, we optimized the harvest time for the P0 TGEV S pseudovirus. The results demonstrated that harvesting 24 h post-transfection yielded the highest titer of TGEV S pseudovirus, further confirming that this time point provided the maximum pseudovirus production (**Figure 3D**).

To validate the reliability of these optimized conditions, we used the optimal conditions to package TGEV S pseudovirus and performed TCID_50_ assays on three independent batches of P0 TGEV S pseudovirus. The results showed that the titers of the three batches of P0 TGEV S pseudovirus ranged from 10^6^ to 10^7^ TCID_50_/mL (**Table 2**).

**Table 2:** Different batches of TGEV pseudovirus titers.

Batch	Pseudovirus titers
1	10^6.5^
2	10^6.25^
3	10^6.25^

### 3.4 Verification of neutralization activity of TGEV S pseudovirus and condition optimization results

**Figure 4:**
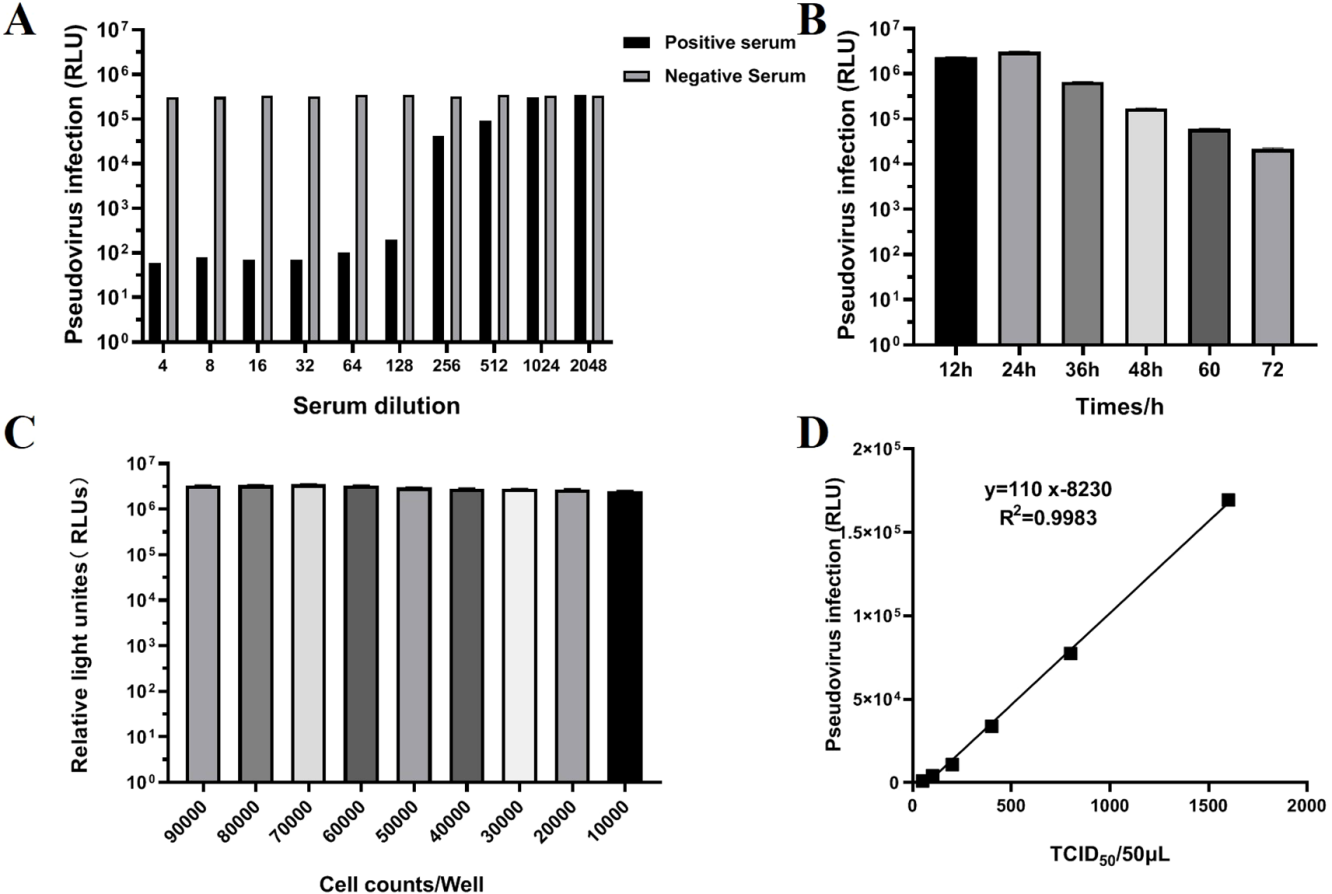
Validation of TGEV pseudovirus neutralization activity and optimization of neutralization assay conditions. **(A)** Validation of TGEV pseudovirus neutralization activity. The test results for positive and negative serum ranged from 1:4 to 1:2048. **(B)** Determination of the optimal detection time (12, 24, 36, 48, 60, 72h) for the TGEV pNT method. **(C)** Determination of the optimal cell inoculation amount (1000, 2000, 3000, 4000, 5000, 6000, 7000, 8000, 9000) using the TGEV pNT method. **(D)** Determination of the optimal TGEV pseudovirus infection level using the TGEV pNT method. The horizontal axis represents the TCID_50_/50 μLof TGEV pseudovirus, and the vertical axis represents the relative light density (RLU) value. The linear equation is: Y=110x-8230.

To verify whether the TGEV S pseudovirus has the ability to be neutralized by TGEV neutralizing antibodies. TGEV-positive and negative sera at different dilution gradients were used to neutralize TGEV S pseudovirus, and the resulting mixture was then inoculated into ST cells. The Relative Luminescence Unit (RLU) value was used as an indicator to assess the infectivity of TGEV S pseudovirus. If the RLU value of the test wells was greater than three times the RLU value of the negative control, it was considered that the pseudovirus successfully infected the cells and was positive. If the RLU value was below this threshold, it was considered negative, indicating that the pseudovirus failed to infect the cells. The results showed that TGEV-positive serum could significantly inhibit the infectivity of TGEV S pseudovirus. As the serum dilution increased, the RLU value gradually increased, indicating that the neutralization effect was enhanced. In contrast, negative serum showed no inhibitory effect and did not significantly affect the infectivity of TGEV S pseudovirus in ST cells (**Figure 4A**). These results confirm that TGEV S pseudovirus can indeed be neutralized by TGEV neutralizing antibodies.

To further optimize the experimental conditions, we screened the time points, cell density, and pseudovirus inoculation doses. The results showed that the optimal detection time was 24 h (**Figure 4B**), during which the cellular response to the pseudovirus was most pronounced. When optimizing cell density in a 96-well plate, a density of 7×10^4^ cells per well exhibited the best pseudovirus infection efficiency (**Figure 4C**). Additionally, when the pseudovirus inoculation dose was 100 TCID_50_/50 μL, the results were the most stable and provided the highest detection sensitivity (**Figure 4D**).

### 3.5 Results of sensitivity, specificity, and stability

**Figure 5:**
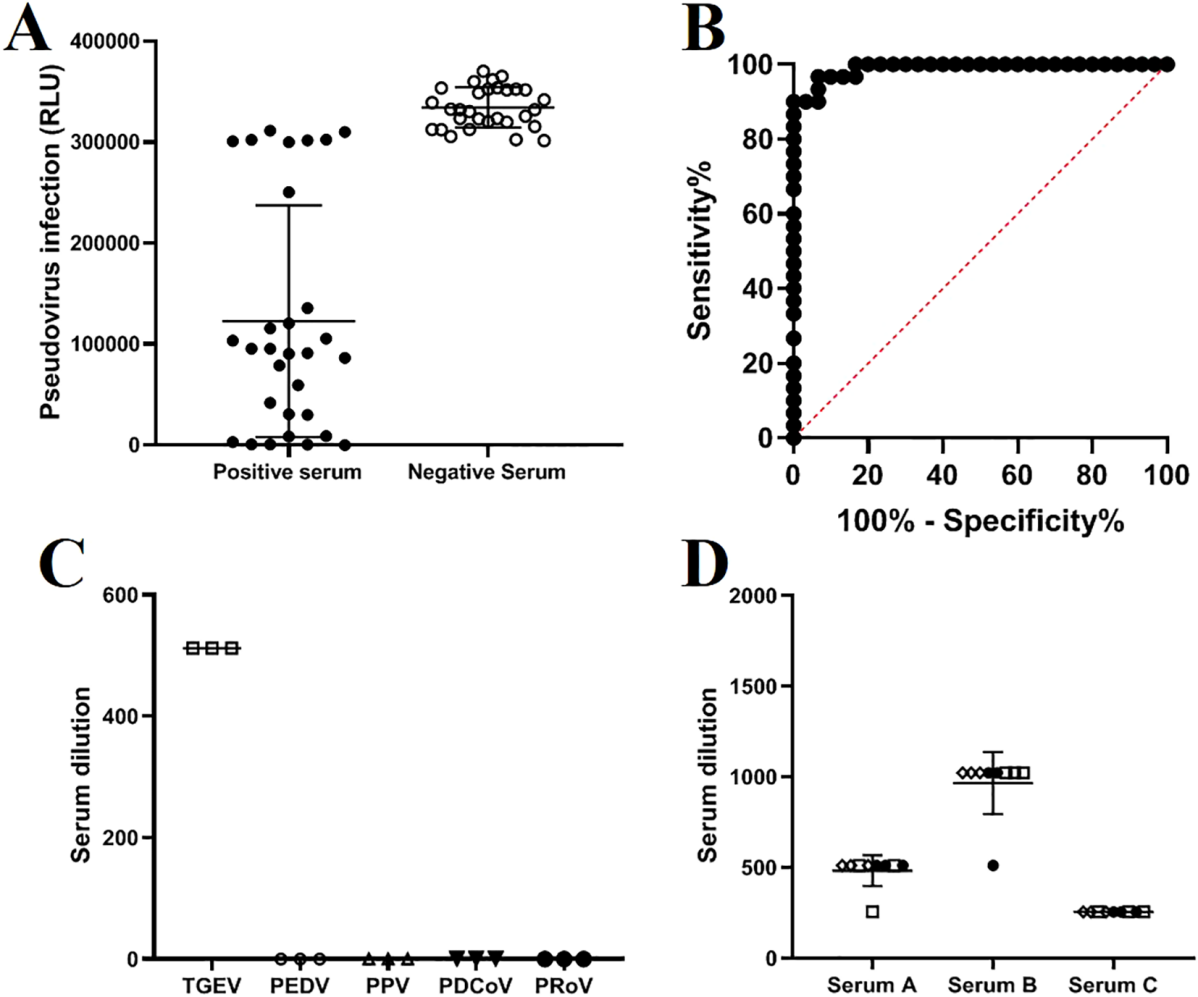
Sensitivity, specificity, and repeatability validation of the TGEV S pseudovirus neutralization assay. **(A)** Results of the TGEV S pseudovirus neutralization assay testing 30 negative and 30 positive serum samples. **(B)** ROC curve analysis of the results from 60 serum samples. **(C)** Cross-reactivity with positive sera from PEDV (1:256), PPV (1:1024), PDCoV (1:512), and PRoV (1:512). **(D)** Intra-batch and inter-batch test results of the TGEV S pseudovirus neutralization assay.

The method established in this study was used to test 30 negative and 30 positive samples. Statistical analysis showed that the sensitivity of the method for detecting serum samples was 100%, and the specificity was 96.6% (**Figures 5A, B**). Additionally, validation using positive sera from viruses associated with pig diarrhea, including PEDV, PPV, and PDCoV, demonstrated that the TGEV S pseudovirus neutralization assay did not exhibit cross-reactivity with these viruses (**Figure 5C**). By analyzing the titers between different batches and within the same batch, the results showed that the titer of each serum sample was similar both within and between batches, with an intra-batch variation of no more than one dilution step and an inter-batch variation of no more than two dilution steps. This indicates that the neutralization assay method is highly consistent and reliable under different experimental conditions, and it can stably assess the neutralization effect of TGEV S pseudovirus (**Figure 5D**). Three positive serum samples were subjected to inter batch and intra batch tests, respectively. The same symbol represents experimental operations performed by the same experimenter.

### 3.6 Concordance comparison results

**Figure 6:**
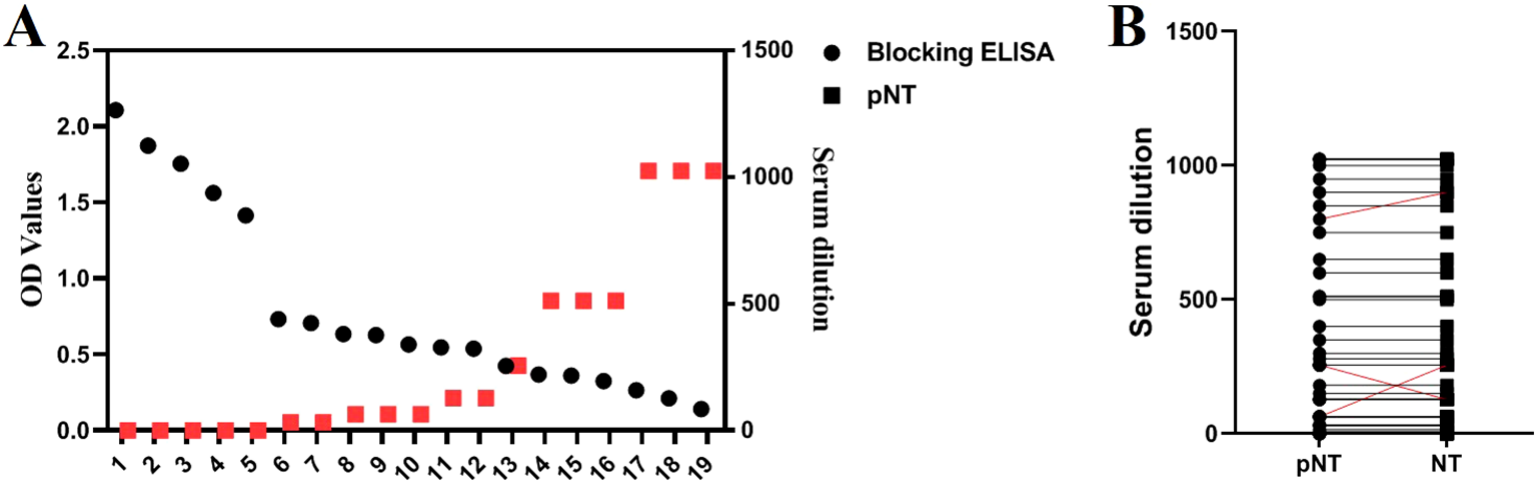
Comparison with Blocking ELISA and TGEV Live Virus Neutralization Assay. **(A)** Simultaneous detection of 19 serum samples using the blocking ELISA and pNT methods. **(B)** Detection results of 50 serum samples using both the TGEV S pseudovirus neutralization assay and the TGEV live virus neutralization assay.

We compared the established TGEV S pseudovirus neutralization assay with a commercial TGEV-blocking ELISA antibody test kit by conducting parallel testing on 14 positive and 5 negative serum samples. The results showed complete consistency between the two methods, with a 100% concordance rate. As the serum titer increases, the OD value detected by the blocking ELISA decreases. From **Figure 6**, it can be seen that there is a high correlation between the antibody titers measured by the blocking ELISA and the pNT method. Correlation analysis shows an absolute value of R = 0.653, indicating a strong correlation (0.61<R<0.8: indicating a strong correlation). In addition, both the TGEV S pseudovirus neutralization assay and the TGEV live virus neutralization assay were used to test 50 pig serum samples. The results showed that the concordance rate for determining serum positivity/negativity was 100%, and the concordance rate for serum titers was 94% (47/50), indicating that the method is highly accurate and holds significant potential for application.

### 3.7 Vaccine efficacy testing results

**Figure 7:**
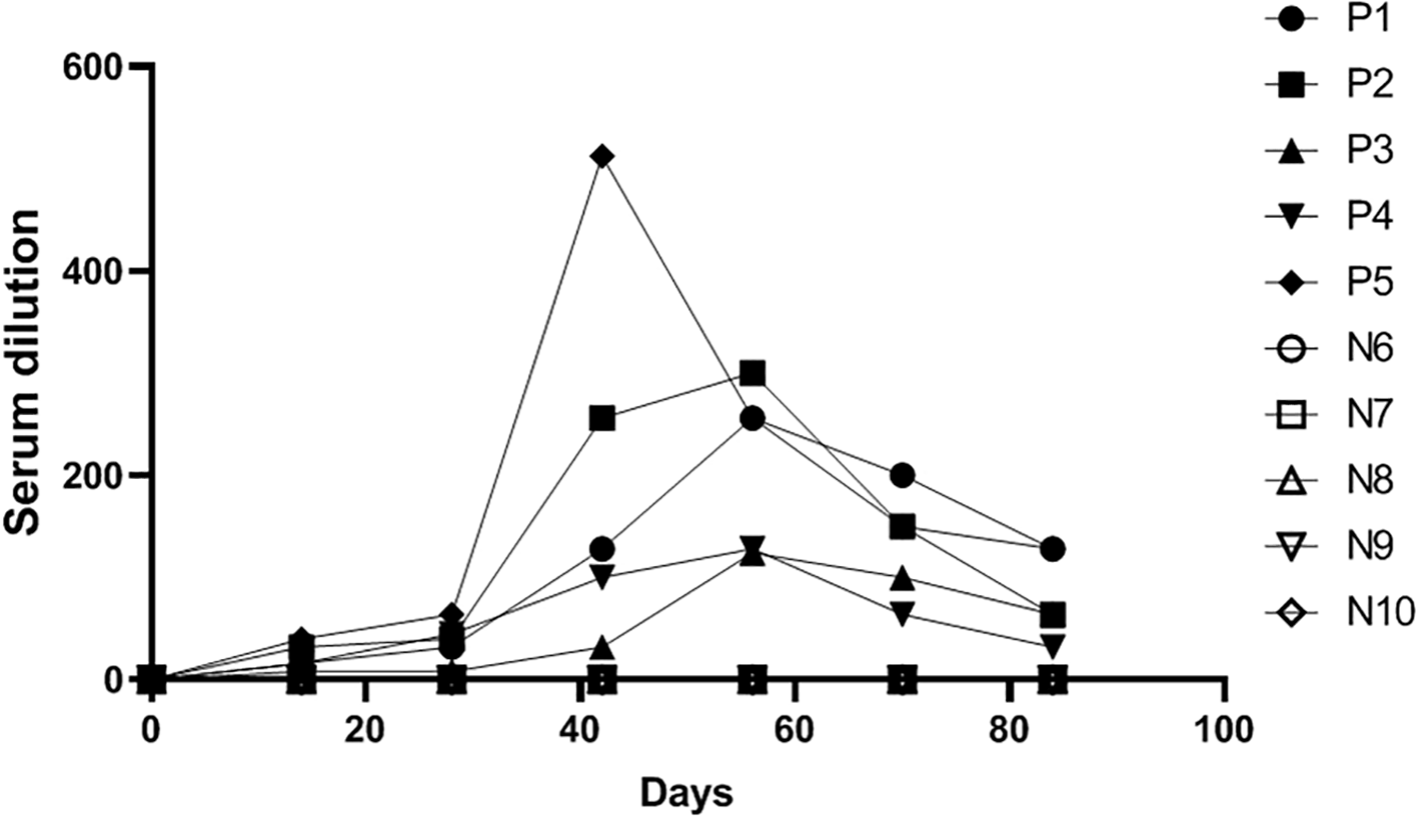
Detection results of serum samples at different time points after TGEV vaccination using the TGEV S pseudovirus neutralization assay.

We collected serum samples from a pig farm at different time points (0, 14, 28, 42, 56, 70, and 84 days) following TGEV vaccination and tested them using the method established in this study to observe changes in antibody levels after vaccination. As shown in **Figure 7**, antibody levels peaked around day 42 and persisted for a prolonged period. Moreover, the results obtained using the established method were consistent with those from the TGEV live virus neutralization assay, indicating that the TGEV S pseudovirus neutralization assay can be used for vaccine efficacy evaluation.

## 4 Discussion

Pseudovirus technology, as an emerging biotechnological tool, has been widely applied in research on important zoonotic pathogens such as SARS-CoV-2 and influenza viruses, especially in the fields of viral infection mechanisms, vaccine development, and neutralizing antibody detection (32, 34, 35). In the vaccine development process, the evaluation of vaccine efficacy is a crucial step, and detecting neutralizing antibody titers and activities is one of the most effective evaluation methods (34, 36, 37). Traditional vaccine evaluation methods often rely on collecting serum from vaccinated humans or animals and conducting neutralization tests with live viruses to assess antibody titers and activities (38). However, this method poses significant operational risks and technical challenges, especially when dealing with highly contagious and hazardous viruses such as influenza, SARS, HIV, rabies, and Ebola, which require research to be conducted in biosafety level BSL-3 or higher laboratories, limiting the efficiency and accessibility of vaccine evaluation (39–42). Therefore, pseudovirus technology, due to its high safety, sensitivity, and reproducibility, has become an ideal tool for vaccine efficacy evaluation (26).

TGEV, a highly contagious coronavirus, poses significant biosafety risks in traditional live-virus research (43). Pseudovirus technology plays a vital role in its study. In TGEV infection experiments, the high transmissibility of live viruses may lead to laboratory contamination (44). Using pseudovirus systems can effectively avoid these issues. The S protein of TGEV is the main surface antigen and is key to its interaction with host cell receptors, directly affecting the virus’s cell tropism and virulence (45, 46). Therefore, the S protein is central to constructing the TGEV pseudovirus system. The VSV system, one of the commonly used systems for pseudovirus construction, is favored for its simple structure, strong replication ability, wide host cell range, and the ability to generate complete viral particles in most animal cells (47, 48). Additionally, this system can allow the incorporation of foreign membrane proteins even in the absence of its own envelope proteins, facilitating easier integration of foreign proteins (47, 48). While the VSV system has the disadvantages of complex operations and longer preparation cycles, it has a higher success rate in packaging, higher viral yields, and faster cell infection efficiency compared to the lentivirus system or other pseudovirus packaging systems (34). Moreover, the pseudoviruses produced by this system better preserve the structure and function of the foreign genes.

In this study, pseudoviruses were successfully constructed using the VSV system, and infection of TGEV target cells ST and PK-15 resulted in the expression of green fluorescence signals, confirming the successful construction of TGEV pseudoviruses. Compared to the lentivirus system, the VSV system not only yielded higher viral titers and infection efficiency but also more accurately replicated the structure and function of the foreign genes. To further verify the function of the pseudovirus, green fluorescent protein (GFP) and luciferase were used as reporter genes to ensure successful packaging (49). If the pseudovirus did not generate significant fluorescence signals in target cells, it would be deemed a failure. Conversely, pseudoviruses with strong fluorescence signals were regarded as successfully packaged and suitable for further analysis. The expression of the reporter genes was closely related to the fluorescence signals (RLU), and RLU values could serve as important indicators for evaluating the neutralizing antibody titers. Compared to the traditional VNT method, which depends on visual observation of cytopathic effects (CPE), the pNT method is more objective and accurate, as it precisely measures RLU values (50). This study found that the TGEV pNT method was directly related to the expression of reporter genes, and the RLU values were used to assess the neutralizing antibody titers. Unlike VNT, which relies on the visual observation of CPE, the pNT method offers a more objective and precise way to determine results. However, pNT is also susceptible to certain interfering factors, such as cell type, cell seeding density, RLU detection time, and pseudovirus sample loading. This study showed that TGEV pseudovirus exhibited infection potential in both ST and PK-15 cells. To determine which cell type was more susceptible to infection, LUC values were measured after infecting ST and PK-15 cells with the TGEV pseudovirus. The results indicated that TGEV pseudovirus infected ST cells most efficiently. Therefore, ST cells were chosen as the target cells for the TGEV pNT. Additionally, cell density was a crucial factor influencing antibody titer determination. Low cell densities resulted in insufficient infection and lower fluorescence signals, while high cell densities caused contact inhibition, reducing the expression of luciferase and affecting the fluorescence intensity. After determining the optimal cell density (7×10^4^ cells per well) and the optimal detection time (24 h post-infection), the pNT method achieved better fluorescence signals. This study significantly shortened the detection time compared to traditional cNT methods, which typically take 3-7 days.

**Figure 8:**
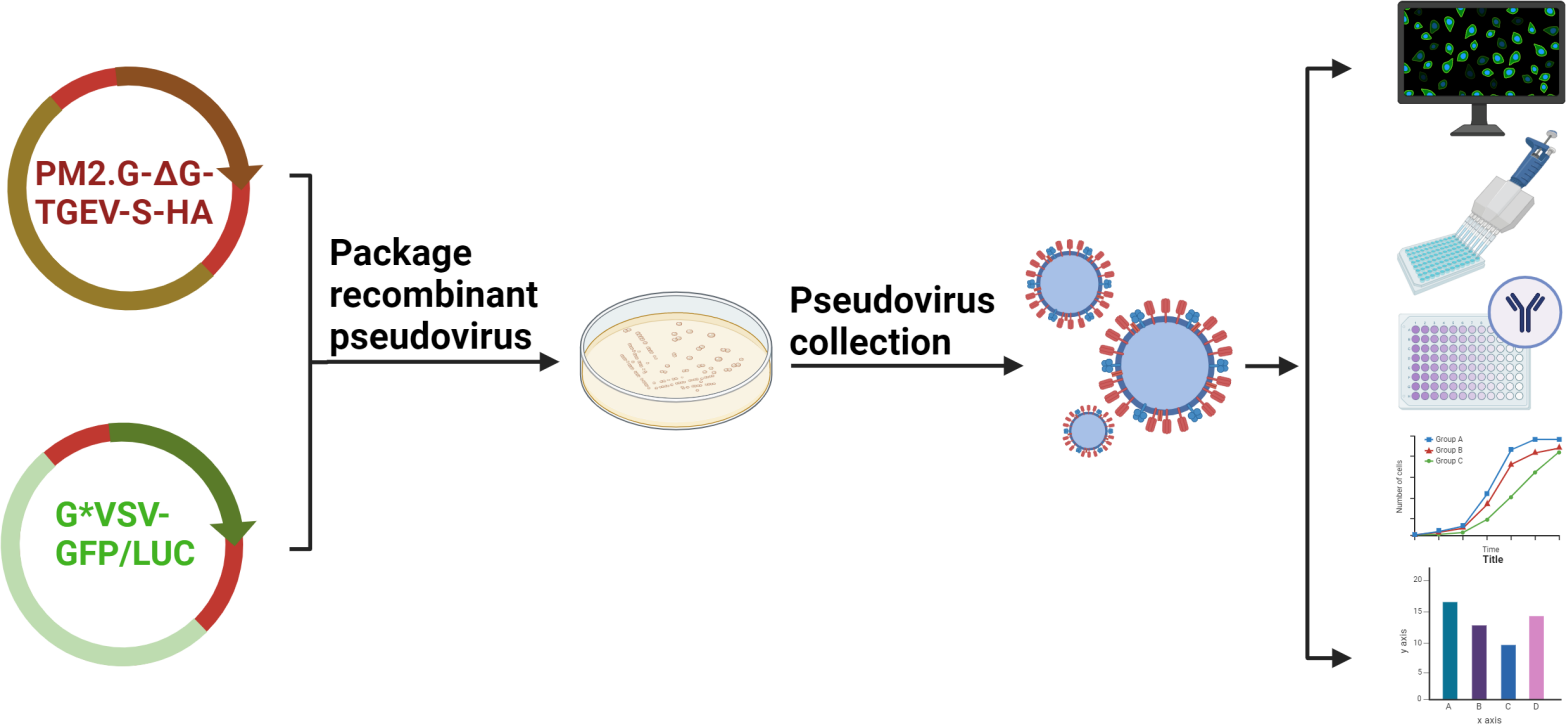
Schematic diagram of the establishment and application of TGEV S pseudovirus packaging and neutralization assay.

The TGEV pNT method was evaluated using the “fixed virus-serum dilution” method, and the end-point neutralization experiment principle was applied. The serum dilution that inhibited 50% of virus infection in host cells was considered the neutralizing antibody titer. The detection results were based on the RLU value, and a positive result was defined as an RLU value lower than half the infectivity of the positive control sample. The virus amount in pseudovirus preparation directly affects serum titer determination, so this study determined the appropriate virus inoculum for the TGEV pseudovirus by evaluating the relationship between pseudovirus infection volume and RLU values. The results showed that RLU values increased proportionally with the pseudovirus titer between 50 TCID_50_/50 μL and 1600 TCID_50_/50 μL. Therefore, within this range, serum neutralization experiments could be performed. To ensure accuracy and reduce pseudovirus usage, this study set the inoculation dose at 100 TCID_50_/50 μL. Finally, before applying the TGEV pNT method in practice, its feasibility and reproducibility were verified. This method showed no cross-reactivity with positive sera of PEDV, PPV, PDCoV, and PRoV. The sensitivity for detecting samples was 100%, and the specificity was 96%. Results indicated that the method had good stability and minimal variation within and between batches. Comparison with ELISA kits demonstrated 100% agreement in detecting positive samples, confirming the potential of the developed method for detecting TGEV infection. However, the sample size was limited, and further validation with more samples is needed to ensure the method’s reliability. Therefore, it is necessary to test more sera or clinical samples to further validate the reliability of this method. In summary, this study successfully constructed TGEV pseudoviruses using the VSV system and optimized and established a pNT detection method. This method provides an efficient, safe, and reliable tool for evaluating the efficacy of TGEV vaccines and detecting serum antibodies. This technology not only overcomes the limitations of traditional methods but also lays a foundation for future pseudovirus research and vaccine development for other infectious diseases (**Figure 8**).

## 5 Conclusion

In this study, the full-length sequence of the TGEV S gene was successfully obtained using segmental amplification and fusion techniques, and it was inserted into the eukaryotic expression vector PM2.G-ΔG-HA to construct the recombinant plasmid PM2.G-ΔG-TGEV-S-HA. The TGEV S pseudovirus was packaged using the VSV system, and its successful assembly was confirmed by inverted fluorescence microscopy, Western blot analysis, and electron microscopy. Packaging conditions were further optimized to achieve high-titer TGEV pseudovirus. Cell tropism analysis revealed significant tropism of the pseudovirus for ST cells, and neutralization experiments demonstrated that TGEV-positive serum effectively inhibited pseudovirus infection of ST cells, confirming its serum-neutralizing activity. A pseudovirus neutralization test (pNT) was developed based on the TGEV pseudovirus, and the conditions affecting its establishment were optimized. Evaluation of the method’s repeatability and detection efficiency showed that the inter-assay and intra-assay errors were both within two dilution steps. Additionally, the consistency rate between this method and a commercial ELISA kit was 100%, indicating good repeatability and high accuracy.

This study successfully constructed a high-titer TGEV S pseudovirus using the VSV system and developed a stable, specific, and accurate pNT method. This method provides a reliable tool for clinical diagnosis, vaccine efficacy evaluation, antibody level monitoring, and studies of infection mechanisms related to TGEV.

## Data Availability

The original contributions presented in the study are included in the article/supplementary material. Further inquiries can be directed to the corresponding author.
